# Analysis of research status and trends on nano-agricultural application: a bibliometric study

**DOI:** 10.3389/fpls.2025.1530629

**Published:** 2025-02-17

**Authors:** Xing Luo, Jing Li, Song Guo, Hua Yu, Xiangzhong Zeng, Zijun Zhou, Yuxian Shangguan, Mingjiang He, Yiting Ouyang, Kun Chen, Zhimin Chen, Yusheng Qin

**Affiliations:** ^1^ Institute of Agricultural Resources and Environment, Sichuan Academy of Agricultural Sciences, Chengdu, China; ^2^ Sichuan Institute of Edible Fungi, Sichuan Academy of Agricultural Sciences, Chengdu, China; ^3^ Soil and Fertilizer Station of Liangshan, Liangshan Yi Autonomous Prefecture Bureau of Agriculture and Rural Affairs, Xichang, China

**Keywords:** nanomaterials, bibliometric analysis, crops resistance, phyllosphere process, sustainable agriculture

## Abstract

**Introduction:**

The rapid global population growth and limitations of traditional agricultural practices have resulted in inadequate nutrient supply. Nano-agricultural technology presents significant potential for enhancing crop growth and resistance, reducing stresses, and providing economic benefits with lower environmental risks.

**Methods:**

In this study, a bibliometric analysis of nano-agricultural applications was conducted using the Web of Science Core Collection, and 2,626 publications from 2000 to 2023 were identified, with an exponential increase in both publications and citations.

**Results and discussion:**

European and Asian countries and institutions are more actively involved, although USA produces the highest-quality papers. Additionally, this field has evolved through two stages: the first stage (2000-2016) focused on the toxicology of nanomaterials (NMs), while the second stage (2017-present) emphasizes NMs as nanofertilizers to promote crop growth, and as nanoregulators or nanopesticides to enhance crop resistance against biotic stress and abiotic stress. Finally, future research perspectives were also proposed, including the optimalizations of NMs, the investigations of the behavior and bioavailability of NMs driven by rhizosphere and phyllosphere process, interdisciplinary collaboration across various fields, the application of NMs from laboratory to the field, and the long-term environmental behaviors and assessments of NMs in diverse ecosystems. Overall, this bibliometric study provides a valuable reference for understanding the development of this field and pinpointing research frontiers.

## 1 Introduction

The global population has surpassed 8 billion in 2022, and related forecasts indicate that the global population will surpass 9.7 billion by 2050 according to the report of United Nations (Jain et al., 2023). Hence, it was forecasted that the global demand for crop production in 2050 will increase by 50-80% (Lowry et al., 2019). Under current agricultural technology conditions (such as improved seeds, fertilizers, and agrochemicals), the global grain production in 2050 will reach 3.1 billion tons, but the above-mentioned grain production still falls short of providing sufficient nutrition for the global population (Alexandratos and Bruinsma, 2012). Additionally, the efficiency of traditional agricultural technologies has gradually declined, and the global grain production growth trend has slowed down since 2000 (Hu et al., 2022; Lowry et al., 2019). Furthermore, the reduced arable land area, water scarcity, climate change, decreased crop stress resistance, and exacerbated environmental degradation will all pose serious threats to global food security (Adisa et al., 2019; Lowry et al., 2019; Zhao et al., 2022). Therefore, the development of alternative traditional agricultural technologies with high utilization, low risk, and sustainable use has become a current research focus and challenge in the international community.

In recent years, with the rapid development of nanotechnology, engineering nanomaterials (NMs) with small size, large surface area, high chemical activity, and high biological effectiveness have provided possibilities for the development of safe, efficient, and sustainable agricultural technologies (Buriak et al., 2022; Lowry et al., 2019). Wang et al. (2020b) reported that the growth-promoting efficiency of nano-fertilizers is 20-30% higher than traditional fertilizers. Wang and White (2022) found that NMs had 43% lower toxicity to non-target pathogens while ensuring their disease control effects compared to the traditional pesticides. Furthermore, various NMs with loading bio-probes (including DNA, antibodies, and nucleic acid aptamers) have also been found to enable rapid and convenient diagnosis of crop diseases compared to traditional plant disease detection methods, ultimately contributing to the prevention and control of crop diseases (Farooq et al., 2021). All above results indicate that NMs have shown promising effects in promoting crop growth and controlling crop diseases. Recently, an increasing number of NMs have been applied in agricultural management and production. For example, Kah et al. (2019) demonstrated that the number of patents granted regarding NMs agricultural application grew exponentially between 1990 and 2016, and yield 3,279 patents filed and 1,254 granted since 1990; Mishra et al. (2017) also found that the investment of nanotechnology in global agriculture reached 75.8 billion dollars by 2020, resulting in a global economic growth of 3.4 trillion US dollars. Therefore, the enormous application potential and research value of NMs in agriculture have become hot and challenging research topics in the international agricultural and environmental fields.

Currently, a large amount of research on nano-agricultural application has been conducted. Meanwhile, Wang, Zhao, Elmer, and others researchers have conducted comprehensive reviews on the promotion of crop growth and enhancement of crop stress resistance by NMs, which played an enlightening and promoting role in this field (Elmer and White, 2018; Wang et al., 2020b; Zhao et al., 2022, Zhao et al., 2020). However, there are few reports on the analysis of the number of past published papers, journals, affiliated disciplines, authors, authors’ countries and institutions, main research content, and development directions in this field. Unlike traditional reviews, bibliometrics is an interdisciplinary field that uses statistical and mathematical methods to quantitatively analyze information carriers, which helps in identifying and visualizing research trends over time (Liu et al., 2023). Bibliometrics can capture thematic networks by mapping the relationships between various research topics, showing how they interconnect and evolve. Furthermore, it can also reveal collaboration patterns by analyzing co-authorship and institutional affiliations, providing insights into the dynamics of research collaboration across different entities. In summary, the bibliometric approach offers a more comprehensive and objective perspective on the research landscape, making it a valuable tool for understanding the development of a field by leveraging large datasets and statistical techniques (Liu et al., 2023; Xie et al., 2020). In recent years, with the development of computer science and information technology, bibliometric visualization analysis software such as CiteSpace and VOSviewer has been widely used in research in various disciplines, including in environmental science, agronomy, medicine, economics, and others (Cao and Wang, 2022; Chen, 2005; van Eck and Waltman, 2009; Wang et al., 2020a).

This study provided a full bibliometric analysis on nano-agricultural application. To achieve this, Web of Science (WOS) was selected as the literature retrieval platform and 2626 publications on NMs agricultural application from 2000 to 2023 were collected and subjected to bibliometric analysis using WOS Data Analysis System, CiteSpace, VOSviewer, and other literature analysis software. The aims are to 1) clarify the development process of nanotechnology application in agriculture and the contributions of various subjects, journals, and authors; 2) uncover the knowledge frame and thematic networks of researches on NMs agricultural application over the past 20 years; 3) illustrate the current research status and future development trends in this field. The findings of this paper will provide guidelines for the future directions of nano-agricultural application.

## 2 Materials and methods

### 2.1 Data collection

In this paper, WOS was chosen as the literature search platform and “WOS Core Collection (SSCI and SCI-EXPANDED)” was used as the literature search database. The search terms were “TS=nano*” and “agriculture application”, the language was English, the document types were “Article” or “Review”, and the publication date ranged from “2000-01-01” to “2023-10-13”. A total of 2,633 articles were retrieved, and 6 retracted publications were removed. A total of 2,627 papers were obtained by selecting original research papers plus reviews, and exported from the database. After checking by HistCite Pro 2.1, one duplicate paper was removed, and a total of 2,626 literature records were eventually obtained.

### 2.2 Bibliometric analysis

The number of publications and citations were obtained from the “analyze search results” function of WOS, as well as publication proportions and publication output of top 10 disciplines according to previous study (Pranckutė, 2021).

As an exceptional visualization software for bibliometric analysis, VOSviewer enables the creation of collaborative network diagrams that illustrate the relationships among authors, institutions, and countries, as well as co-occurrence network diagrams featuring keywords (van Eck and Waltman, 2009). Thus, the collaborative network diagrams of authors, institutions, and countries were displayed by VOSviewer (v. 1.6.19) in this paper. Subsequently, co-occurrence network diagrams of keywords were also performed by VOSviewer and the minimum number of occurrences of keywords were set as 36. Note that some non-technical words (e.g., “acid”, “oxide”, and “I;”) were ignored because of their meaninglessness according to previous method (Yu et al., 2024). Moreover, the size of the circles represents the number of papers, and total link strength (TLS) quantifies the connections between a node and all others, including repetitions, which emphasizes frequently co-occurring or closely related nodes in visualizations. For example, if a keyword often co-occurs with several others, its TLS will be higher, resulting in a thicker line in the chart.

The intermediary centrality of top 10 categories, burst detection of key words, and co-citation of publications were analyzed by CiteSpace (v. 6.2.4) (Wang et al., 2022). The number of publications from 2000 to 2009 was 40 and there was no burst detection of key words. Hence, the range of burst detection of key words and co-citation of publication were both performed from 2010 to 2023. As the basic indicators of bibliometrics, total local citation score (TLCS) is represented as the number of citations of a journal in local databases (databases composed of exported literature), and total global citation score (TGCS) is defined as the number of citations of a journal in global databases, which provides an overall indication of the impact and influence of the journal within the academic and research community (Li et al., 2022). Thus, TLCS and TGCS were conducted by HistCite Pro 2.1 as described by Shah et al. (2020).

## 3 Results

### 3.1 Evolution and distribution of publications related to NMs on agriculture application

**Figure 1:**
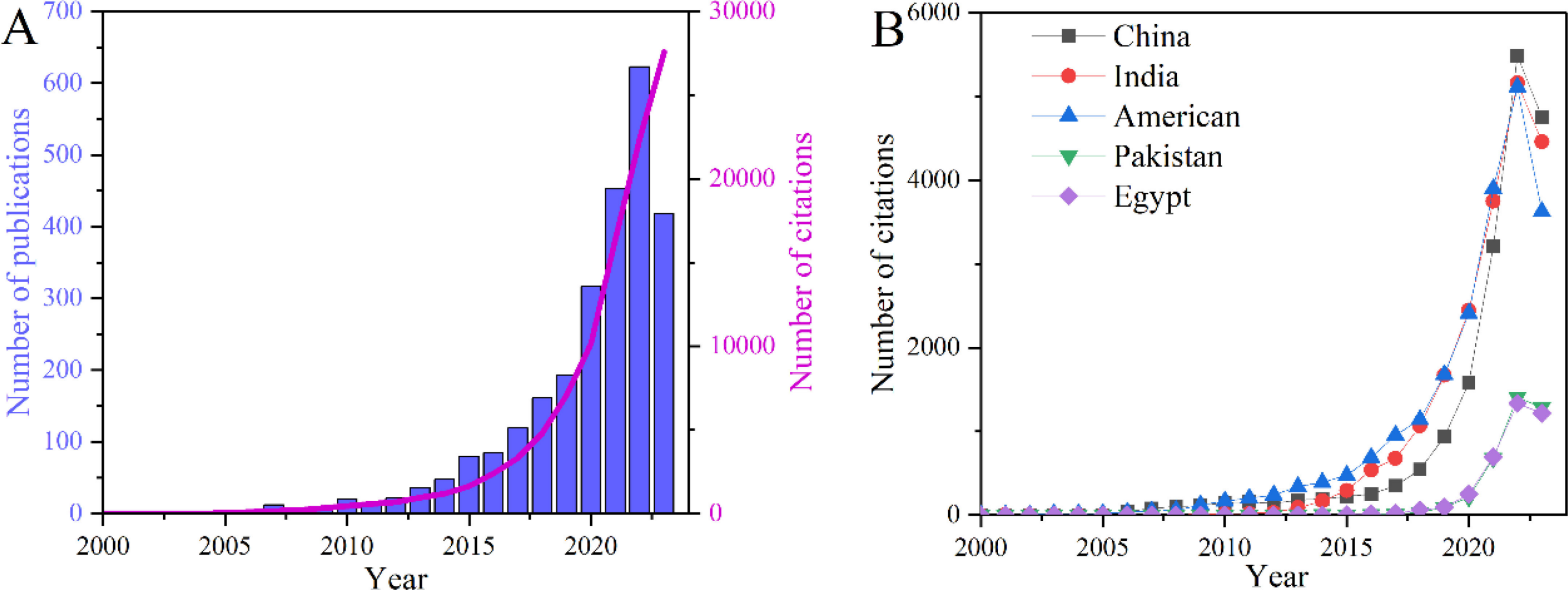
The trend of the number of publications and citations **(A)** and the change of the citation frequency in the main issuing countries **(B)** regarding nanomaterials (NMs) on agriculture application from 2000 to 2023 (Source of articles data: Web of Science).

First, a total of 2626 papers were published from 2000 to 2023 based on WOS (**Figure 1A**). The first paper published at 2000 and there were only 40 papers and 448 citations appeared at the first decade (2000-2009). After the first ten years development, the number of publications and citations has shown a rapid growth trend (**Figure 1A**), indicating that the agricultural application of NMs has been a hotpot and attracted much attention by scientists. The top five countries in terms of the number of publications were China (727), India (598), The United States of America (USA) (373), Pakistan (156), and Egypt (140). However, when it comes to total citations, the top five countries are currently USA, India, China, Pakistan, and Egypt (**Figure 1B**). To be specific, Chinese researchers have published the highest number of papers (727) and the number of citations of these papers was 18,479, leading to an average citation per publication of 25.42. Meanwhile, researchers from the United States have published a total of 373 papers with a total citation of 21,628, and the average citation *per* publication were 57.99. More specifically, the trend of the total citations among top five countries followed the order of USA (21,628) > India (20,389) > China (18,479) > Pakistan (3,727) > Egypt (3,684) (**Figure 1B**). Hence, both of the total citation and the average citation *per* article ranked first for USA. Although China ranked first in terms of the number of publications, it ranks third in total citations and fourth in average citation, indicating there were still a certain gap compared to other countries.

**Figure 2:**
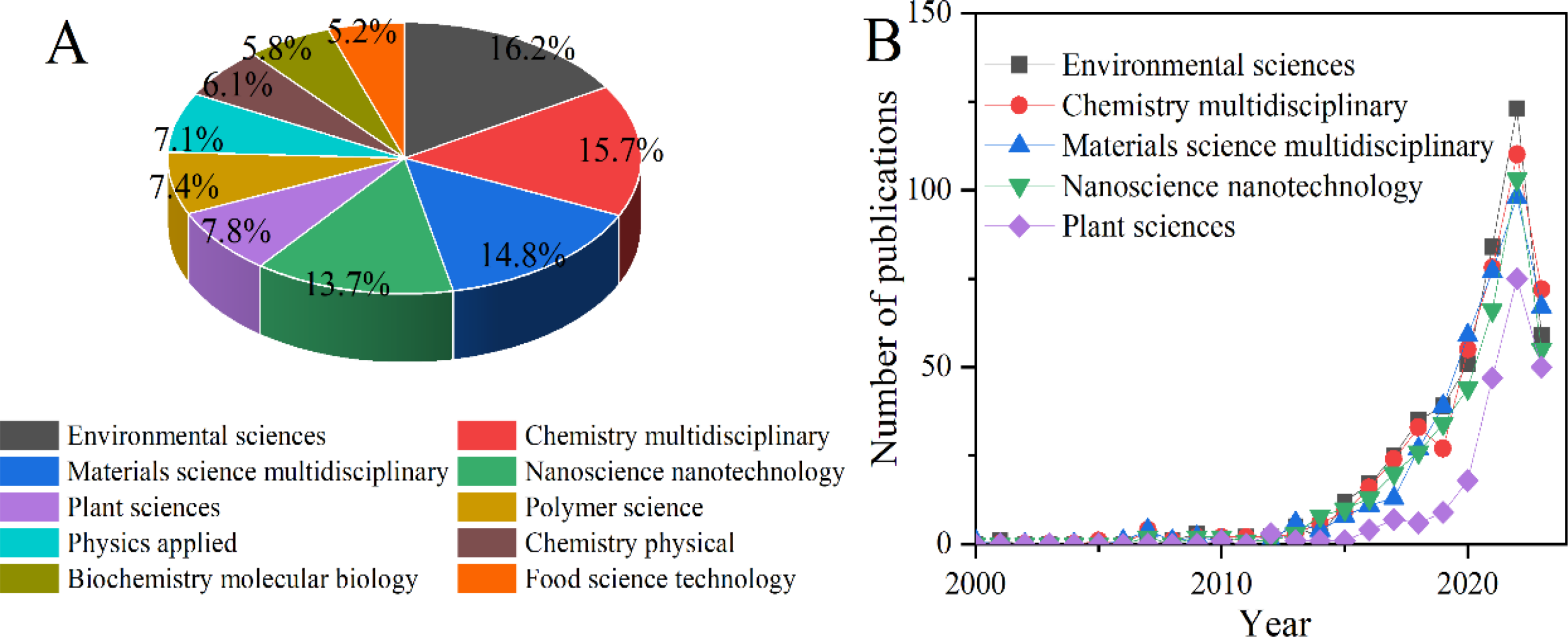
Publication proportions **(A)** of the top ten disciplines and publication output **(B)** of the top five disciplines during 2000-2023 in the field of NMs agricultural application.

The publication proportions of the top ten disciplines were analyzed according to WOS data analysis system and shown in the **Figure 2A**. The results showed that top five disciplines related to the application of NMs in agriculture are Environmental science (16.2%), Chemistry multidisciplinary (15.7%), Materials science multidisciplinary (14.8%), Nanoscience nanotechnology (13.7%), and Plant sciences (7.8%) (**Figure 2A**). Similarly, the number of publications in the top five disciplines also exhibited a continuous growth trend (**Figure 2B**), consistent with the overall number of publications mentioned in the previous section. The intermediary centrality in disciplines can be used to characterize the interdisciplinary nature of that discipline with other disciplines (Goldman, 2014). The intermediary centrality in top ten disciplines were analyzed by CiteSpace software and the results showed that the discipline with the highest intermediary centrality was Biochemistry molecular biology (0.21), while the lowest were Plant sciences (0.04) and Physics applied (0.04) (**Table 1**). Notably, the intermediary centrality of the top 4 disciplines all exhibited a good intermediary centrality, and ranged from 0.13 to 0.19. Although the fifth discipline (Plant science) has certain advantages in revealing the mechanism of nanoparticles and plants, it had a lower intermediary centrality of 0.04. Hence, more integrations with other disciplines should be expanded and strengthen for Plant sciences in the future research.

**Table 1:** Top 10 disciplines based on the number of published papers and intermediary centrality regarding nanomaterials (NMs) on agriculture application from 2000 to 2023.

Category	Intermediary centrality
Environmental science	0.19
Chemistry multidisciplinary	0.13
Materials science multidisciplinary	0.19
Nanoscience nanotechnology	0.15
Plant sciences	0.04
Polymer science	0.11
Physics applied	0.04
Chemistry physical	0.09
Biochemistry molecular biology	0.21

HistCite Pro 2.1 was used to analysis the main journals of all publications and the results showed that top three journals in this field were “Nanomaterials” (58 articles), “Science of The Total Environment” (52 articles), and “Journal of Agricultural and Food Chemistry” (41 articles) (**Table 2**). Among them, the third-rank journal “Journal of Agricultural and Food Chemistry” not only had the highest TLCS (320), but also had the highest citation frequency *per* paper (59.12). In addition, journals in the top discipline (Environmental science) also exhibited a good growth trend (**Table 2**). For example, the classical journal “Science of The Total Environment” in the discipline of Environmental science not only had the highest TGCS (2,793), but also possessed the highest impact factor (IF) among the top 10 journals in terms of publications (IF2018-2023 9.6); another classic journal “Environmental Science: Nano” has published 35 papers, and ranked third in IF (IF2018-2023 7.9) and TLCS (189).

**Table 2:** Top 10 journals in terms of the number of published papers in the field of NMs agricultural application from 2000 to 2023.

Journal	Number of published papers	(2018-2023) Impact factor (2018-2023)	Total local citation score (TLCS)	Total global citation score (TGCS)	Citation frequency per paper
*Nanomaterials*	58	5.4	0	1339	23.09
*Science of The Total Environment*	52	9.6	197	2793	53.71
*Journal of Agricultural and Food Chemistry*	41	6.3	320	2424	59.12
*Environmental Science and Pollution Research*	38	5.4	59	816	21.47
*International Journal of Biological Macromolecules*	38	7.8	90	1316	34.63
*Environmental Science: Nano*	35	7.9	189	818	23.37
*Frontiers in Plant Science*	35	6.8	0	629	17.97
*Scientific Reports*	35	4.9	0	1333	38.09
*Chemosphere*	34	8.3	4	1021	30.03

### 3.2 Collaborative network diagrams of contributing countries, institutions, and authors

**Figure 3:**
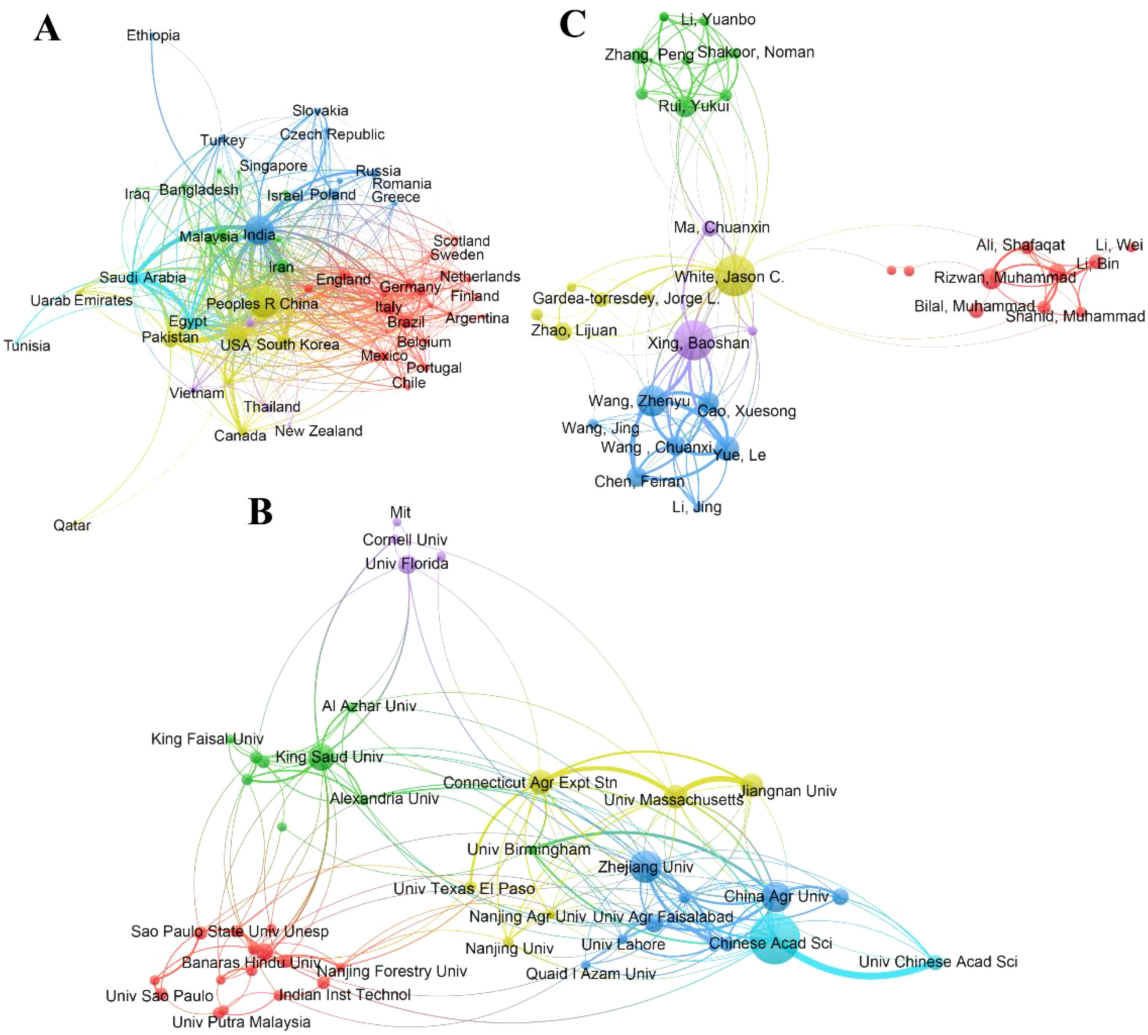
Cooperation among main paper contributor countries **(A)**, institutions **(B)**, and authors **(C)** regarding NMs on agriculture application from 2000 to 2023.

The collaborative relationships between countries were analyzed by VOSviewer software. A total of 105 countries or regions contributed and 52 countries are displayed on **Figure 3A** based on the minimum number of publications of a country at 5. Among the top 52 countries, the trend of different continents in the top 52 countries followed the order of Europe (21 countries) > Asia (20 countries) > Americas (6 countries) > Africa (3 countries)> Oceania (2 countries). TLS can be used to represent the strength of collaboration between different countries, and collaboration between countries is beneficial to achieve technological innovation and improve research level (Mao et al., 2020). The trend of TLS among top five countries followed the order of China (497) > India (485) > USA (397) > Saudi Arabia (248) > Pakistan (235). To be specific, TLS between China and USA was 105, followed by China and Pakistan (43), and China and India (61). Given above findings, China exhibited the best performance in collaborating with the main publishing countries.

With respect to the research institutes, 3,235 organizations were identified according to VOSviewer and 48 organizations are presented in **Figure 3B** based on the minimum number of publications of an organization at 13. Chinese Academy of Science is the leading institution with 89 publications, followed by Zhejiang University (53), China Agricultural University (49), King Saud University (44), Connecticut Agricultural Experiment Station (39), and University of Massachusetts (38) (**Figure 3B**). And the trend of top 5 research institutions in terms of TLS values followed the order of Chinese Academy of Sciences (56) > China Agricultural University (49) > University of Massachusetts (46) > Connecticut Agricultural Experiment Station (44) > Zhejiang University (44) (**Figure 3B**). In addition, Jiangnan University and Chinese Academy of Agricultural Sciences from China also ranked 8th and 9th respectively, with TLS values at 27. Given above findings, organizations from China and USA both exhibited certain advantage in this field. Notably, it is also worth noting the average citation frequency of other Chinese research institutions among the top 9 research institutions in terms of TLS values, except for the Chinese Academy of Sciences, were all lower (17.65-28.47) than that of related research institutions in the United States, Saudi Arabia, and Pakistan (35.52-63.41).

From the perspective of author, a total of 12,651 authors contributed and top 63 authors are showed in **Figure 3C** with more than 6 publications. The top three authors are White Jason C from Connecticut Agricultural Experiment Station, Xing Baoshan from University of Massachusetts, and Wang Zhenyu from Jiangnan University (**Figure 3C**). And all above researchers have published more than 20 papers. Meanwhile, Wang Zhenyu exhibited the greatest value in TLS, followed by Xing Baoshan (90), Yue le (84), and Cao Xuesong (78). In addition, White Jason C not only exhibited good performance in TLS (68), but also presented the highest value in term of the total citations (1,937) and the citation frequency (60.53) (**Figure 3C**). Moreover, although Gardea-torresdey jorge (The University of Texas) has only published 10 papers, he has received 1,196 citations, as well as Zhao Lijuan (Nanjing University) (13 papers, 1,187 citations) and Rui Yukui (China Agricultural University) (16 papers, 567 citations).

### 3.3 Research progress of NMs on agriculture application

**Figure 4:**
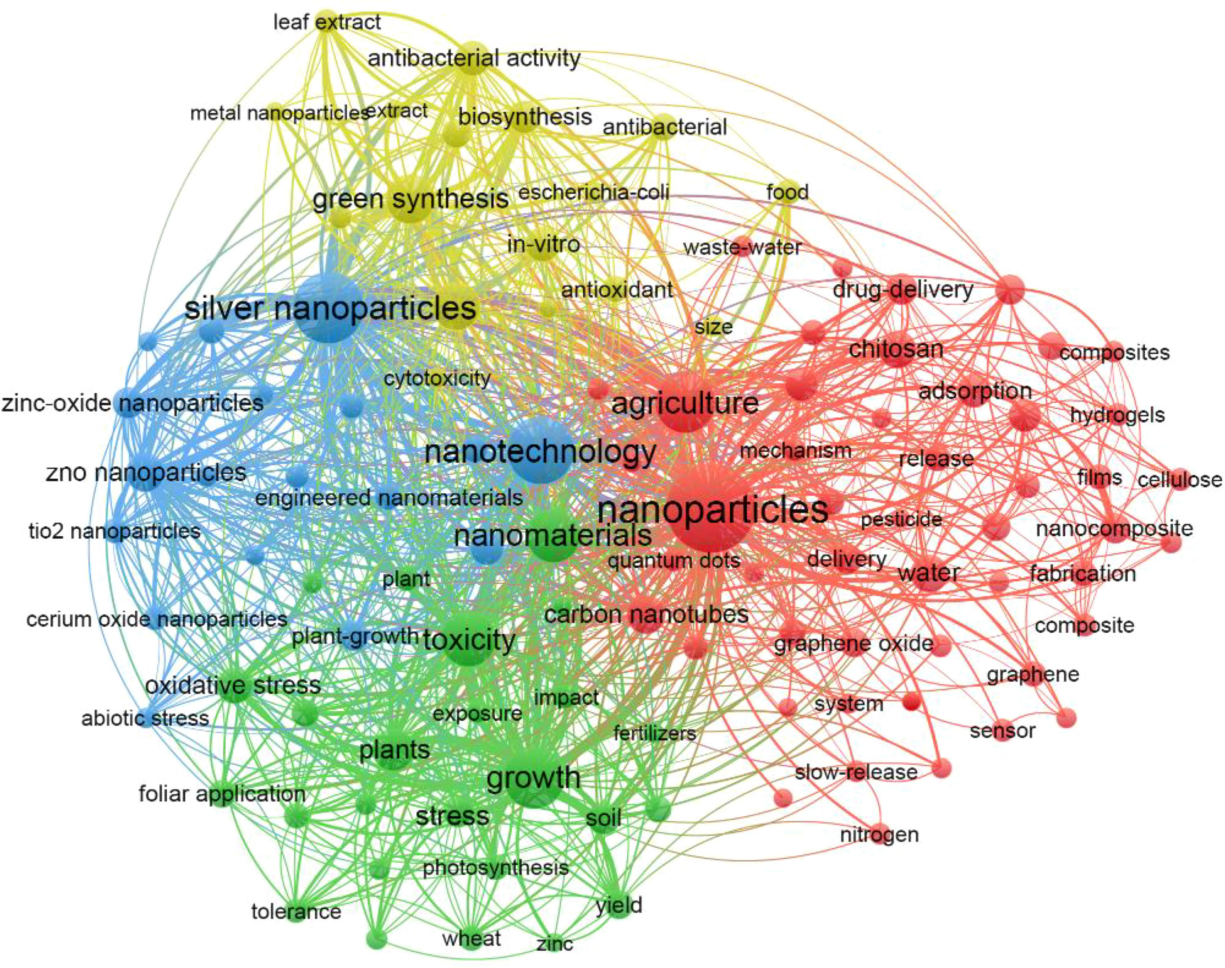
Keyword co-occurrence network during 2000-2023 in the field of NMs agricultural application.

The keywords are the precise expression of the publication’s topic, and the frequency of keyword occurrence is closely related to the attention of the research topic (Zhu et al., 2021). Therefore, the co-occurrence network of keywords was conducted by VOSviewer. A total of 12,131 keywords are involved in this topic and top 107 keywords are displayed in **Figure 4** based on the minimum number of occurrences of a keyword at 36. In **Figure 4**, the node size represents the number of co-occurrences, and the colors reflect different clusters classified according to co-occurrence analysis. The top 10 keywords with the highest frequency of occurrence are “Nanoparticles”, “Silver Nanoparticles”, “Nanotechnology”, “Growth”, “Agriculture”, “Toxicity”, “Nanomaterials”, “Green synthesis”, “Gold nanoparticles”, and “Plants”. Meanwhile, the trend of the TLS in the top 10 keywords also followed the same order (**Figure 4**). Moreover, the frequency of occurrence of “ZnO nanoparticles”, “Antibacterial activity”, “Oxidative stress”, and “Carbon nanotubes” are greater than 100, and their TLS values are also at a high level (> 490). As for research topic analysis, all keywords were classified into 4 different clusters by the VOSviewer software (**Figure 4**). Cluster 1 (red color) is mainly involved in the physical and chemical behavior of non-metallic NMs and the applications of NMs, including using as the delivery materials and sensors. Keywords such as chitosan, carbon nanotubes, graphene, adsorption, release, sustained release, delivery, and sensors are recognized in this section. Cluster 2 (yellow color) mainly focuses on the green synthesis of NMs and their application in bacteria inhibition, and the major keywords are green synthesis, biosynthesis, antibacterial activity, and cytotoxicity. Cluster 3 (blue color) mainly includes various typical metallic NMs, such as silver nanoparticles, zinc oxide nanoparticles, titanium dioxide nanoparticles, and cerium oxide nanoparticles. Cluster 4 (green color) mainly investigates the response mechanism between different NMs and plants. In this cluster, the first section focuses on the toxic mechanism of NMs on plants, and the keywords includes toxicity and oxidative stress; the other section mainly includes the application of NMs as new fertilizers to promote plant growth and as regulators to enhance crop resistance against stress, and the keywords contains foliar application, tolerance, photosynthesis, fertilizer, and yield.

**Figure 5:**
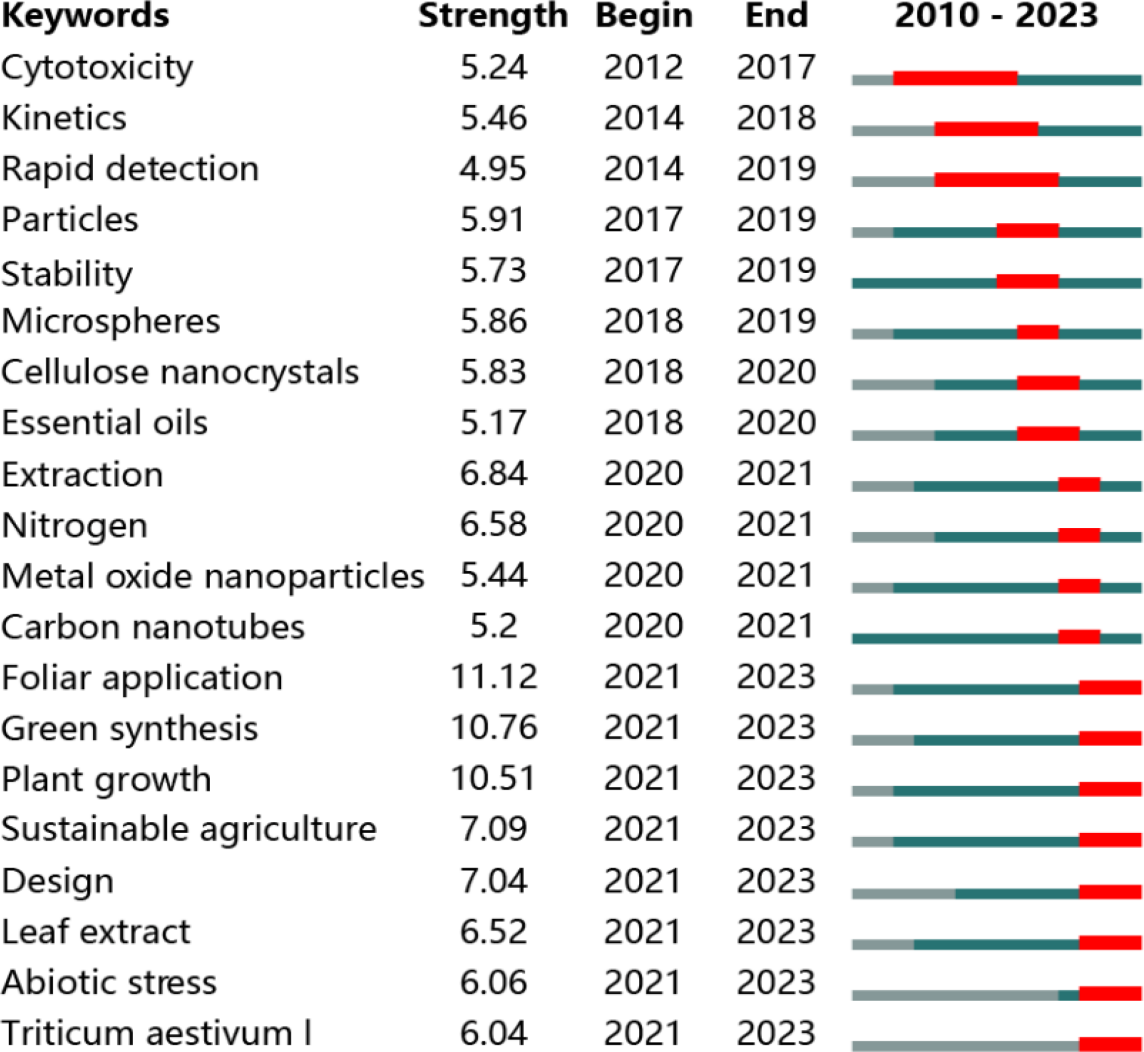
Burst detection of top 20 keywords from the published papers in the field of NMs agricultural application from 2010 to 2023.

The burst detection of keywords was performed to illustrate the evolution of a certain research field in a period of time by CiteSpace according to Lin et al. (2020) and shown in **Figure 5**. Due to the small number of published papers in the first 10 years, the burst detection of keywords from 2000 to 2009 was ignored. The results showed that “Cytotoxicity” “Kinetics” and “Rapid detection” exhibited high strength in the early period from 2010 to 2023. Subsequently, the keywords of “Cellulose nanocrystals” “Metal oxide nanoparticles” and “Carbon nanotubes” were highly studied. Of particular note is that the top three keywords with strongest citation bursts were “Foliar application” “Green synthesis” and “Plant growth”, which became research hotspots starting in 2021 and have continued until now. Furthermore, other keywords related to plant growth, such as “Sustainable agriculture” “Abiotic stress” and “Triticum aestivum 1 (wheat)”, as well as the keywords related to green synthesis like “Leaf extract”, all started to become research frontiers and maintained research interest till now. Hence, the changes of keywords indicates that the research process of NMs on agriculture application has evolved from the toxicology study of NMs on bacteria and plant to the positive regulation of NMs on crop growth and the enhancement of crop tolerance against stress. Additionally, Wang et al. (2020b) found many NMs (such as CeO_2_, Cu, CuO, Fe_3_O_4_ and ZnO NMs) exhibited a trend of “low-promoting-high-inhibiting” on the interaction between NMs and plants; Cao and Wang (2022) also reviewed that low concentration NMs not only showed great potential in promoting crop growth, but also exhibited great performance in activating crop resistance against stress, which are consistent with our above findings.

### 3.4 The most co-cited publications

**Figure 6:**
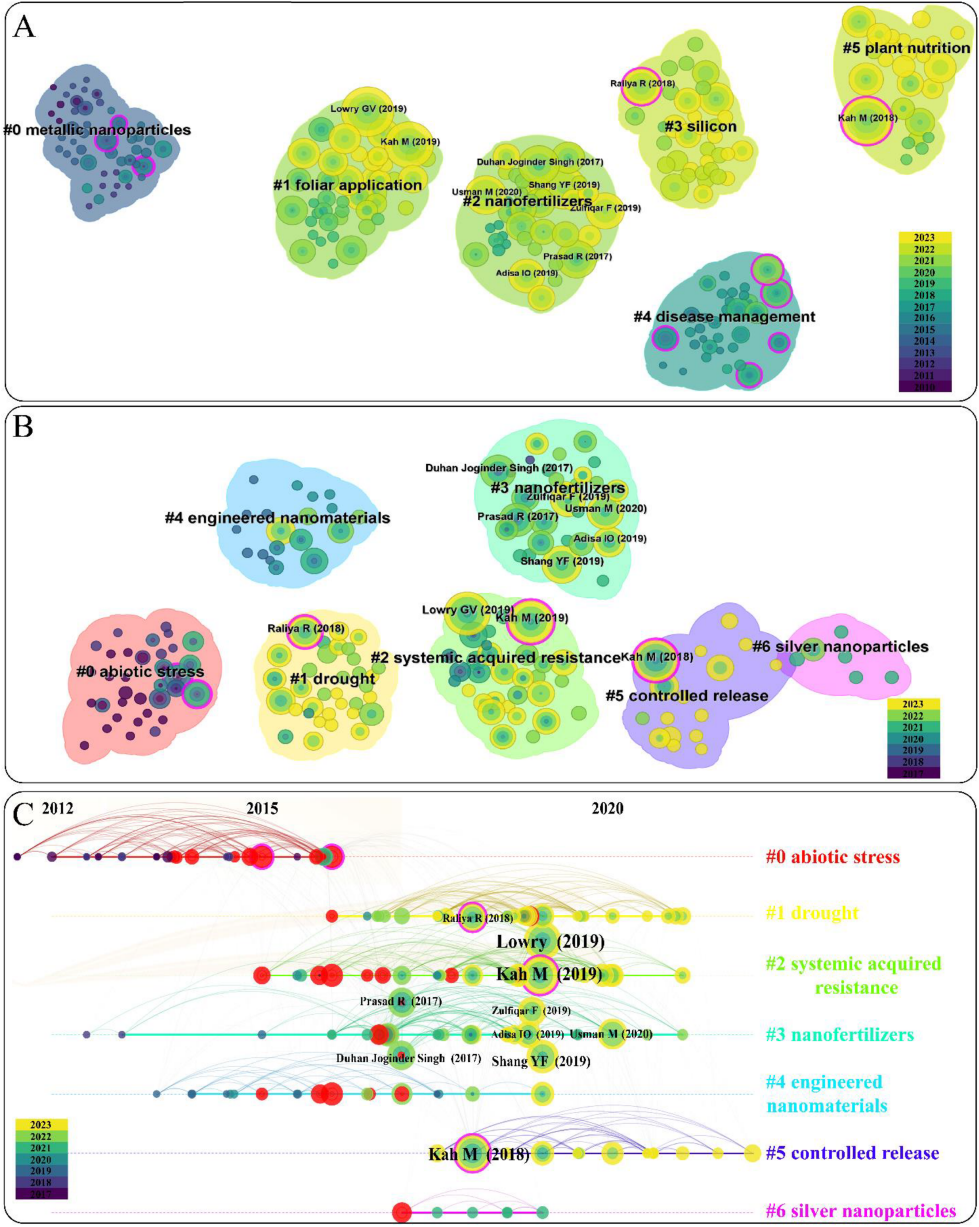
Graphs of co-citation of publication on NMs agricultural application from 2010-2023 **(A)** and 2017-2023 **(B)**. Time series charts of co-citation of publication on NMs agricultural application from 2017-2023 **(C)**.

Co-citation analysis is a method used in bibliometrics to examine the relationship between two publications that are both cited in the reference list of a third citing publication (Donthu et al., 2021). This analysis is commonly employed to uncover the quantitative characteristics and intrinsic laws of scientific development (Zhu et al., 2021). Hence, the co-citation analysis of NMs on agriculture application was performed in CiteSpace and displayed in **Figure 6**. Firstly, the publications from 2000 to 2009 were also ignored and a total of 28 clusters were identified from 2010 to 2023. And top six clusters (251 nodes, Q = 0.5231) were chosen and shown in **Figure 6A**. The top six clusters are metallic nanoparticles (0) (49 publications), foliar application (1) (43 publications), nanofertilizers (2) (39 publications), silicon (3) (35 publications), disease management (4) (33 publications), and plant nutrition (5) (27 publications). Interestingly, it can be observed that four clusters (Cluster 1, 2, 3, and 5) have emerged as research hotspots in recent years based on the color distribution. Notably, the dates of top ten co-cited publications all begin from 2017. Hence, the co-citation analysis from 2017 to 2023 was deeply analysised.

The total number of identified clusters are 19 from 2017 to 2023 and the top seven clusters (184 nodes, Q =0.4288) are below: abiotic stress (0) (34 publications), drought (1) (34 publications), systemic acquired resistance (2) (32 publications), nanofertilizers (3) (31 publications), engineered nanomaterials (4) (19 publications), controlled release (5) (16 publications), and silver nanoparticles (6) (6 publications) (**Figure 6B**). As known, drought is one kind of abiotic stress. Hence, cluster 0 and cluster 1 were organized into the same cluster (abiotic stress) in this paper. Thus, abiotic stress, systemic acquired resistance, and nanofertilizers are the top hotpots of NMs on agriculture application. Specifically, the main research areas of top three clusters are also listed: (1) the application of NMs (such as silicon nanoparticles) to increase plant water use efficiency against abiotic stress; (2) foliar application of NMs (such as carbon dots) promote plant systemic acquired resistance and antioxidative system; (3) the application of NMs as nanofertilizers to increase plant nutrient use efficiency.

The top ten co-cited publications are listed at **Figures 6B-C** and **Table 3** and the results showed that cluster 1 has one publication, cluster 2 has two publications, cluster 3 has six publications, and cluster 5 has one publication. Notably, the top two co-cited publications are both involved in cluster 2 (systemic acquired resistance). To be specific, the top one is from Lowry et al. (2019) and published in nature nanotechnology, which receives 99 citations *per* year (doi: 10.1038/s41565-019-0461-7). It systematically illustrated the great potential of NMs on agriculture application, including improving the use efficiency of water, light, and agrochemicals, strengthening soli integrity and function through microbiome enhancements, and augmenting crop resistance against environmental stressors. Finally, it also discussed the challenges to overcome to realize the full potential of NMs agricultural application, such as the interaction and mechanisms of NMs-plant, the methods to improve the efficiency of NMs into plants. The other impactful publication is published by Kah et al. (2019) with the average citation of 90 (doi: 10.1038/s41565-019-0439-5). This paper not only reviewed the great potential of nano-enabled strategies to improve crop nutrition and protection, but also emphasized the importance of the field experiments to expand the use of NMs on agriculture application. The third co-cited publication is identified in cluster 5 (controlled release), published by Kah et al. (2018), and received over 400 citations since 2018. It reviewed the benefits of NMs as nanofertilizers to improve crop growth and nanopesticides to enhance crop resistance compared to their conventional analogues. Although the publications in cluster 3 had not been the top three in the co-cited publications, it had the highest number of the publications, indicating the application of NMs as nanofertilizers have been attracted a lot of attentions by global scientists. Overall, it is concluded that the main areas of interest in this field currently focus on the use of NMs as nanofertilizers to enhance crop growth and as regulators to improve crop resistance, consistent with our above results.

**Table 3:** Details of the top 10 co-cited papers in the local database on NMs agricultural application from 2010-2023.

Author	Title	Publication time	Frequency	Total citations	Citations per year	DOI
Lowry et al. (2019)	Opportunities and challenges for nanotechnology in the agri-tech revolution	2019	113	396	99	10.1038/s41565-019-0461-7
Kah et al. (2019)	Nano-enabled strategies to enhance crop nutrition and protection	2019	107	360	90	10.1038/s41565-019-0439-5
Kah et al. (2018)	A critical evaluation of nanopesticides and nanofertilizers against their conventional analogues	2018	102	462	92.4	10.1038/s41565-018-0131-1
Shang et al. (2019)	Applications of nanotechnology in plant growth and crop protection: A review	2019	84	322	80.50	10.3390/molecules24142558
Usman et al. (2020)	Nanotechnology in agriculture: Current status, challenges and future opportunities	2020	73	277	92.33	10.1016/j.scitotenv.2020.137778
Ram et al. (2017)	Nanotechnology in sustainable agriculture: Recent developments, challenges, and perspectives	2017	72	437	72.83	10.3389/fmicb.2017.01014
Duhan et al. (2017)	Nanotechnology: The new perspective in precision agriculture	2017	71	378	63.00	10.1016/j.btre.2017.03.002
Raliya et al. (2018)	Nanofertilizer for precision and sustainable agriculture: Current state and future perspectives	2018	66	249	49.80	10.1021/acs.jafc.7b02178
Zulfiqar et al. (2019)	Nanofertilizer use for sustainable agriculture: Advantages and limitations	2019	65	222	55.50	10.1016/j.plantsci.2019.110270
Adisa et al. (2019)	Recent advances in nano-enabled fertilizers and pesticides: A critical review of mechanisms of action	2019	58	229	57.25	10.1039/C9EN00265K

## 4 Discussion and future research perspectives

### 4.1 Cooperations promote the development of nano-agricultural application

This paper has not only facilitated the enhancements of understanding on subjects, trends, and channels of nano-agricultural technology, but also garnered an overview and implications of the global research in this field. Web of Science Core Collection (SCIE and SSCI) was selected as the data source, as it is frequently accessed in this type of research due to its multidisciplinary character, and it could also supply comprehensive data and necessary indicators for determining the citations and impacts of academic works (Aleixandre-Tudó et al., 2020). Thus, the terms (nano and agriculture application) were utilized to understand the development of nano-agricultural application thoroughly based on WOS.

The first bibliometric analysis of nanotechnology applications was published in 1997 (Aleixandre-Tudó et al., 2020; Braun et al., 1997). It not only stated the advantages of nano-structured materials over bulk materials, but also stressed the that nanotechnology was an emerging area of research. Although there was no content of nano-agricultural technology in the first bibliometric study, the similar rapid development occurred in nano-agricultural application. According to the number of publications and citations, the development trend of NMs agricultural application could be divided into two stages, the first stage is the preliminary exploration stage and ranged from 2000 to 2009; the second stage (from 2010 till now) is the rapid growth stage, and 98.17% papers has been published in this stage (**Figure 1A**). Worth of note is that the development trend of different countries and discipline all followed the similar trend (**Figures 1B**, **2**), indicating the topic of NMs agricultural application is gaining more attention and more resources are being invested in this field.

Although global scientists are aware of the importance of nano-enabled strategy, the levels of development are still uneven between different regions and different countries. European countries rank first in the top number of countries participating in this project (21 countries, 40.38%), followed by Asia (20 countries, 38.46%), America (6, 11.54%), Africa (3, 5.77%), and Oceania (2, 3.85%) (**Figure 3A**). On the other hand, Asian countries not only account for four of the five in the top number of publications (**Figure 1B**), but also account for four of the five highest TLS countries (**Figure 3A**), indicating Asian countries exhibited good performance in publishing papers, as well as collaborating with other publishing countries. The similar trend occurred in the top 10 research institutions that Asian institutions account for 70 percent of the top ten research institutions (**Figure 3B**). A recent study reported that Asia continues have the highest number of people suffering from hunger, with 402 million individuals representing 55 percent of the global undernourished population in 2022 (FAO et al, 2023). This may clarify why Asian countries have played a key role in publishing and participating in international cooperation efforts in this field. Moreover, more than 38 percent (282 million) of undernourished people live in Africa (FAO et al, 2023), but Africa only has three countries (South Africa, Nigeria, and Ethiopia) was lined in the top 52 countries in the number of publications, which may be due to their relatively low economic level. To be specific, the two most populous countries in the world from Asian (China, 727 publications, and India, 598 publications) are very enthusiastic about the research of nano-agricultural technology, and this phenomenon should also be driven by the need to ensure the food security of their countries. Similarly, as the world’s largest grain export country, USA also recognized the importance of nano-agricultural application at the beginning of this field and has achieved a better leading role in this field. USA not only has two of the top three most influential authors, but also accounts for 40% of the top ten co-cited papers (**Figures 3C**, **6C** and **Table 3**). In addition, national academies of sciences, engineering and medicine in USA also jointly released a research report on “ Science breakthroughs to advance food and agricultural research by 2030”, pointing out that nanotechnology is one of the important ways to break through the current bottlenecks in agricultural technology and make plants better able to cope with environmental challenges (including heat, cold, drought, floods, pests and nutritional needs), which also greatly promote the development of nano-agricultural applications (National Academies of Sciences, Engineering, and Medicine, 2018).

The outstanding contributors to this field based on the number of publications are White Jason C, Xing Baoshan, and Wang Zhenyu (**Figure 3C**). Among them, White Jason C is the most influential because of the highest total citations and the highest citation frequency. Although they all make great contributions to the development of this area, they have different focuses. White Jason C firstly published several fundamental reviews, including two of the top 10 co-cited papers (Nano-enabled strategies to enhance crop nutrition and protection and Recent advances in nano-enabled fertilizers and pesticides: A critical review of mechanisms of action), which played foundational roles in the development of this area. White Jason C also systematically studied the effects and mechanisms of copper-based NMs on crop disease inhibition. White Jason C and his colleagues firstly explored the effect of foliar application of six NMs (B, CuO, MnO, SiO_2_, TiO_2_, and ZnO NMs) against fusarium wilt, the results indicated that CuO NMs exhibited the best performance in alleviating the damage of watermelon disease in greenhouse experiments and field pot experiments (Elmer et al., 2018). Subsequently, White Jason C and his colleagues stressed the importance of particle morphology, composition and dissolution behavior for copper-based NMs on crop disease. For example, they found that foliar application of Cu_3_(PO_4_)_2_·3H_2_O NMs at a concentration of 10 mg/L was significantly more effective in suppressing crop wilt compared to CuO NMs at a concentration of 1000 mg/L. This superior effectiveness can be attributed to the unique nanosheets morphology, the presence of phosphorus, and the higher dissolution rate of Cu_3_(PO_4_)_2_·3H_2_O NMs (Borgatta et al., 2018). White Jason C and his colleagues also clarified the related mechanisms of Cu-based NMs against crop disease, such as Cu-based NMs could increase the level of antioxidants and upregulate the relative expression of disease-related genes, thus finally results in the enhancements of system acquired resistance against crop disease (Borgatta et al., 2018; Elmer et al., 2018; Ma et al., 2020). Although Wang Zhenyu had no paper in the top 10 co-cited papers, he had been participated in 23 publications and ranked the top one in TLS (94) (**Figure 3C** and **Table 3**). Professor Wang Zhenyu not only majored in the research and development of nano-fertilizers and nano-pesticides, but also paid much attention to the rhizosphere processes of NMs. Wang emphasized that rhizosphere processes, such as root secretions, microbial and earthworm activities, could enhance the bioavailability of NMs and increase the uptake of NMs by plant, thus finally maximizing the potentials of NMs applications for boosting food crop production and global food security (Wang et al., 2020b). As shown in **Figure 3C**, professor Xing Baoshan is in the middle of the two above researchers, mainly because he participated in most of their work. For example, professor Xing not only discussed the importance of rhizosphere processes for NM with professor Wang, but also anticipated in exploring the mechanism of copper-based against crop disease with professor White Jason C (Ma et al., 2020; Shang et al., 2020; Wang et al., 2020b). Therefore, effective scientific collaboration plays a crucial role in the advancement and development of global science.

### 4.2 The comprehensive understanding of the toxicology of NMs promotes the application of NMs in agriculture

The analysis of the keywords indicated that the development of NMs in agriculture application was divided into four different clusters (**Figure 4**). However, the development of the timeline in this field is absent in **Figure 4**, and the analysis of the burst detection of keywords was subsequently performed, which filled the above-mentioned shortcomings. By analyzing the two images together, it can be observed that the nano-agricultural application has gone through two development stages (**Figures 4**, **5**). The first stage is the toxicological study of NMs on the health of different species, including human, mammal, plant, microorganisms, and cells. In 2626 screened publications, Scott (2005) firstly highlighted the transformative potential of nanotechnology in agriculture and food systems, including disease treatment delivery systems, new materials for pathogen detection, new tools for molecular and cellular biology, protection of the environment, and the security of agricultural and food systems (Scott, 2005). However, he also cautioned that there were potential unforeseen risks that may accompany with these significant benefits. Therefore, a careful, comprehensive, and balanced evaluation of the risks of nanotechnologies were urgently needed at the initial stage of nano-agricultural application. Subsequently, Singh and Nalwa (2007) immediately stated the toxicity and risk assessments of nanostructured materials on human health; Ray et al. (2009) also critically discussed the fate, behavior, and toxicity of various classes of NMs in the environment. These important reviews mentioned above are part of the 48 screened publications from 2000 to 2009 included in this paper, and they had played an important role in promoting the subsequent development of the toxicology of NMs. As shown in **Figure 4** and **Figure 5**, keywords involved in the toxicology of NMs, such as “Cytotoxicity” (ranged from 2012 to 2017), “Kinetics” (ranged from 2014 to 2018) and “Rapid detection” (ranged from 2014 to 2019), were highly studied during the early period from 2010 to 2023. This reinforces the importance of toxicological research of NMs as the first stage in the application of nano-agricultural.

After a substantial amount of toxicological research on NMs, there is growing interest in how to utilize NMs for the development of sustainable agriculture. The keywords related to the application of NMs in sustainable agriculture, such as “Foliar application”, “Green synthesis”, “Plant growth”, “Sustainable agriculture”, “Abiotic stress”, and “Triticum aestivum 1 (wheat)”, have all become research frontiers since 2021 and continue to be relevant today (**Figures 4**, **5**). Moreover, the top 10 co-cited articles shared a common theme of the sustainable application of NMs, and most of them have also been published within the last five years (**Figure 6** and **Table 3**). Therefore, above findings revealed that the second stage is the utilization of NMs in sustainable agriculture application. Zhao et al. (2020) demonstrated that some NMs with unique physiochemical properties inherently enhance plant growth and stress tolerance, than merely acting as nano-carriers. The main methods for NMs entering crop included seed dressing, root soaking, soil application, and foliar application (Luo et al., 2023; Ma et al., 2021; Cao et al., 2021). The application of various NMs, such as carbon NMs, selenium NMs, silicon NMs, iron NMs, and zinc NMs have shown great potential in promoting crop growth as nanofertilizers (Cao et al., 2022; Cheng et al., 2023; Singh et al., 2013; Wang et al., 2021; Yue et al., 2023). In addition, the application of various NMs, such as copper NMs, manganese NMs, carbon NMs, selenium NMs, silicon NMs, and iron NMs have also shown great potential in enhancing crop resistance against different stress as nanoregulators or nanopesticides (Cai et al., 2020; El-Shetehy et al., 2020; Lu et al., 2020; Luo et al., 2021; Ma et al., 2020; Zhou et al., 2021). Hence, there is a growing diversity of family members composed of various types of NMs in this field of sustainable agriculture. Furthermore, present literatures also revealed the relevant mechanisms of NMs on crops at some extent. For example, nanofertilizers enhanced crop growth by slowly supplying nutrient and increasing nutrient use efficiency; nanopesticides alleviated biotic stress by direct antimicrobial and insecticidal property, activation of host resistance for pathogens and pests, and alteration of host metabolism; nanoregulators could trigger the secretion of reactive oxygen species in plants in advance to stimulate crop defense systems (including antioxidant enzymes, such as superoxide dismutase, catalase, peroxidase, ascorbate peroxidase, glutathione S-transferase, and antioxidant metabolites, such as ascorbic acid, glutathione, oxidized glutathione), ultimately enhancing the resistance of crops (Zulfiqar et al., 2019; Wang et al., 2020a; Cao et al., 2022; Zhao et al., 2022; Cao et al., 2024).

Worth of note, keywords like “Rapid detection” related to the use of NMs as nanosensors have been a prominent research area from 2014 to present (**Figure 5**). While nanosensors have been applied in human medicine for the past decade, reports on the use of nanosensors in agriculture are relatively rare (Zhang et al., 2024). Nanosensors can not only contribute to promoting sustainable food production by enabling the early detection of pathogens, fertilizers, herbicides, pesticides, moisture, diseases in crops and animals, the presence of heavy metal ions, and toxins, but also assist in measuring diverse parameters such as soil pH, chlorophyll content, photosynthetic content, protein content, and the total nutrient uptake (both macronutrients and micronutrients) by plants (Bharti et al., 2024). In addition, smart agriculture practices, which involve the utilization of advanced technologies including artificial intelligence (AI), the internet of things, machine learning, and cloud computing, possess the potential to bring about a revolutionary change in agricultural nanosensors by offering more efficient quality assessment. For instance, AI-driven nanosensors with the assistance of Global Positioning System could precisely guide agricultural irrigation by measuring the total soil moisture content (Bharti et al., 2024). With the implementation of these advanced nanosensors, farmers can boost crop yields, optimize fertilization techniques, and conserve resources through the detection and measurement of specific nutrients.

### 4.3 Future research perspectives of nano-agricultural application

**Figure 7:**
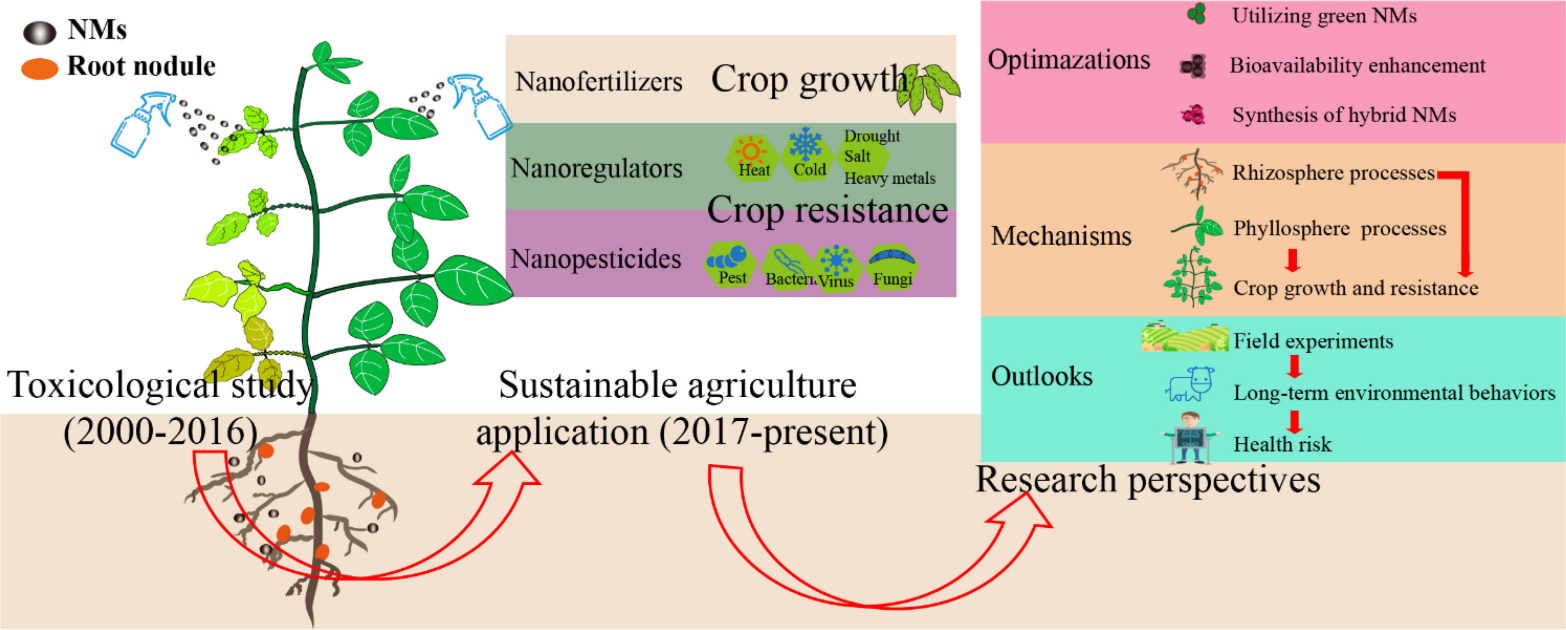
The development and perspectives of nano-agricultural application.

Numerous studies indicate that nano-agricultural technology not only exhibits better performance in promoting crop growth and enhancing crop resistance than that conventional analogues do, but also offers greater sustainability, and has fewer detrimental effects on the environment and human health (Cao and Wang, 2022; Wang and White, 2022). Nonetheless, there is significant potential for further enhancement. For example, Cao et al. (2021) reported that the disease control efficacy of 1 mg/plant of green sulfur NMs increased infected tomato biomass by 143% than that of a commercial fungicide (Hymexazol); Anwaar et al. (2024) discovered that green synthesis of Fe_2_O_3_ NMs could not only increase potato growth, but also exhibited a concentration-dependent inhibitory in the severity and incidence of potato early blight disease; Luo et al. (2023) demonstrated that polyvinylpyrrolidone coated La_2_O_3_ NMs increased the bioavailability of NMs, thus notably decreasing cucumber wilt by 67.62% as relative to the infected control; Wang et al. (2023) subsequently demonstrated the fresh tomato biomass in the treatment of hybrid LaPO_4_ NMs was increased by 60% than that of La_2_O_3_ NMs; Wang et al. (2021) indicated that nitrogen-doped carbon dots (CDs) increased the light conversion and electron supply of undoped CDs, finally increasing crop photoelectron transfer rate, and improving the corn yield by 24.50%. Hence, the optimalization of NMs, including utilizing green or green synthetic NMs, bioavailability enhancements, and synthesis of hybrid NMs shows significant potential in advancing nano-agricultural applications and warrants further investigations (**Figure 7**).

Soil application and foliar application are two main application methods for NMs into crop. Rhizosphere is where plant roots, soil, and soil biota interact, and the rhizosphere processes could alter the bioavailability of nutrients and NMs, ultimately enhancing crop production (Wang et al., 2020b). Zhao et al. (2016) demonstrated that the exposure of Cu NMs (10 mg/L) significantly increased the content of root exudates, and reduced solution pH in hydroponic nutrient solution. The elevated proton concentration further stimulated the release of Cu ions. Rhizosphere microorganisms are regarded as the second genome of plants and play an important role in promoting crop growth and resilience (Kumar and Dubey, 2020). Jiao et al. (2023) reported that soil application of Se NMs (0.1 mg/kg) enhanced rice seedling growth by increasing synthesis and secretion of organic acids, which resulted in attracting more colonization of *Bacillus* and *Pseudomonas* in the rhizosphere. In addition, Maet al. (2021) also found that the combined addition of 10 mg/kg SiO_2_ NMs and earthworms enhanced the content of silicic acid in the rhizosphere by 11.5%, and improved the abundance of silicate solubilizing bacteria and the bioavailability of Si in the rhizosphere, finally increasing maize growth and yield. Over all, the potential and related mechanisms of the behavior and bioavailability of NMs driven by rhizosphere processes, including root secretions, microbial and soil fauna activities, have been explored and discussed at some extent. However, foliar application of NMs have attracted much attentions as it ranked second cluster in the analysis of co-citation (**Figure 6A**, and **Figure 7**). Recent researches also indicated that foliar application has certain advantages compared to traditional soil application. First, NMs for foliar application could be absorbed more quickly into crop leaves and transported more rapidly to the parts of the crop that required, thereby enhancing their functionality (Hong et al., 2021); Second, the amount of NMs needed for foliar application is relatively lower, thus significantly reducing the environmental burdens (Avellan et al., 2021). In addition, Song et al. (2021) found that foliar application of ZnO NMs can alleviate low-temperature stress in rice, and the ability of NMs to mitigate crop stress was positively correlated with the absorption of ZnO NMs. This may be due to the fact that only the NMs entering crops can induce the formation of crop resistance, thereby enhancing the resistance of crop. The absorption of NMs by crops is directly related to the bioavailability of NMs, and the bioavailability of NMs is crucial to perform the functions of NMs (Wang et al., 2020b). For foliar application, the phyllosphere process of NMs is the key process for regulating their bioavailability. Additionally, phyllosphere process of NMs is mainly influenced by the crop leaf organs and the phyllosphere microorganisms. For example, Xiong et al. (2016) found that organic and inorganic mixtures released by phyllosphere microorganisms could accelerate the dissolution of NMs, thereby improving the absorption and transport of relevant nutrient elements in leaves; Ji et al. (2024) also demonstrated that foliar application of 10 mg/L CDs improved the abundance of phyllosphere microorganisms in maize, including *Verrucomicrobia*, *Proteobacteria*, *Actinobacteria*, and *Deinococcus*-*Thermus*, finally result in an increase in fresh weight of maize roots and shoots (54.9 % and 50.5 %). However, it is still not clear how the phyllosphere process regulates the bioavailability of NMs, thus increasing the function of NMs on crop growth and tolerance. Therefore, exploring the impact of the phyllosphere process on the behavior and bioavailability of NMs in agricultural ecosystems is crucial for the effective and safe development of nano-enabled agricultural technologies.

Different and various NMs have shown promise in improving crop growth and enhancing resistance against biotic and abiotic stresses in greenhouse experiments (Wang et al., 2022; Cao et al., 2024). And the effects of NMs on field experiments were also performed at some extent. For example, Elmer et al. (2018) demonstrated that foliar application of CuO NMs produced 53% more watermelon fruit relative to the *fusarium* infected control in the field experiments, which was also superior to the commercial fungicides; Cao et al. (2023) also reported that spray with La_10_Si_6_O_27_ nanorods resulted in increasing rice yield by 35.4% and enhancing seeds nutritional quality relative to the thifluzamide treatment in field experiments. Although NMs have achieved certain effects in some field experiments, the types of NMs tested in these trials are relatively limited, and the scopes of the field experiments are relatively small. Additionally, the effects of NMs on agricultural environment are still mostly limited in small ecosystems, such as mostly in the greenhouse potting soil. For instance, Luo et al. (2023) evaluated that foliar application of 200 mg/L La_2_O_3_ yield the best performance in decreasing cucumber wilt, and the content of La left in soils was 2.75 mg/kg soil based on the greenhouse experiments. The total of La content in potting soil was much lower than critical concentration of La for crops and soil ecosystems. However, the assessments on the long-term effects of NMs in field trials and different food chains are still lacking (Acharya and Pal, 2020; Singh and Gurjar, 2022). Therefore, it is essential to further strengthen the implementation of field trials to assess the effectiveness and long-term environmental behaviors of NMs in agricultural application, particularly in large-scale test sites and mesocosms (**Figure 7**) (Cao and Wang, 2022). Furthermore, the potential human health risks associated with direct and dietary exposure to NMs should also be investigated more thoroughly (**Figure 7**).

## 5 Conclusions

In this present study, the development of NMs on agriculture application exhibited a thriving trend based on the number of publications and citations. Although many countries and institutions in Europe and Asia have contributed in this field, USA holds the first rank in the total citation and the average citation for the publications. The communication and cooperation of nano-enabled agricultural technology still need to be strengthened, such as the exchanges between different disciplines, the cooperation between different countries and research institutions. The evolution of this field has gradually changed from the toxicological study of NMs to some novel research hotpots, the first is used to be as nanofertilizers to promote the growth of crops, and the second is used to be as nanoregulators or nanopesticides to change the resistance of crops against different stress based on the analysis of keywords and co-cited publications. Finally, future research priorities and recommendations are provided below:

1. the optimalizations of NMs could increase the efficacy and bioavailability of NMs. Therefore, the optimalizations of NMs, including utilizing green or green synthetic NMs, bioavailability optimalization, and synthesis of hybrid NMs are warranted;
2. Soil application and foliar application are two main application methods for NMs into crop. Rhizosphere processes (e.g., root exudates and rhizosphere organisms) and phyllosphere processes (e.g., crop leaf organs and phyllosphere microorganisms) can regulate the bioavailability and environmental behavior of NMs. However, the soil and phyllosphere environment are complicated, the mechanisms of the behavior and bioavailability of NMs driven by rhizosphere and phyllosphere process should be performed in greenhouse and field experiments for better understanding the environmental behavior of NMs during agricultural application;
3. Enhancing cooperation among different institutions and authors, and facilitating interdisciplinary integration across plant biology, phytopathology, materials synthesis, microbiology, mathematical modeling, and soil science hold significant importance for sustaining the advancement of NMs applications in agricultural environment, where the mechanisms of NMs on crop growth and resistance should be further explored comprehensively.
4. Various NMs have shown promise in improving crop growth and enhancing resistance in greenhouse experiments. However, the long-term environmental behaviors and evaluations of NMs in field experiments and diverse ecosystems have been rarely investigated. Furthermore, the potential risk that NMs present to human health, regardless of whether the exposure occurs directly or *via* the diet, continue to be aspects that also urgently demand further in-depth explorations.

## Data Availability

The original contributions presented in the study are included in the article/supplementary material. Further inquiries can be directed to the corresponding author.
